# The Potential Role of DNA Methylation in Abdominal Aortic Aneurysms

**DOI:** 10.3390/ijms160511259

**Published:** 2015-05-18

**Authors:** Evan J. Ryer, Kaitryn E. Ronning, Robert Erdman, Charles M. Schworer, James R. Elmore, Thomas C. Peeler, Christopher D. Nevius, John H. Lillvis, Robert P. Garvin, David P. Franklin, Helena Kuivaniemi, Gerard Tromp

**Affiliations:** 1 Department of Vascular and Endovascular Surgery, Geisinger Health System, Danville, PA 17822, USA; E-Mails: jelmore@geisinger.edu (J.R.E.); rpgarvin@geisinger.edu (R.P.G.); dfranklin@geisinger.edu (D.P.F.); 2 Sigfried and Janet Weis Center for Research, Geisinger Health System, Danville, PA 17822, USA; E-Mails: ronning@susqu.edu (K.E.R.); rerdman@geisinger.edu (R.E.); cmschworer@gmail.com (C.M.S.); cdnevius@geisinger.edu (C.D.N.); shkuivaniemi@geisinger.edu (H.K.); gctromp@geisinger.edu (G.T.); 3 Department of Biology, Susquehanna University, Selinsgrove, PA 17870, USA; E-Mail: peelert@susqu.edu; 4 Department of Ophthalmology, Wayne State University School of Medicine, Detroit, MI 48202, USA; E-Mail: johnlillvis@gmail.com; 5 Department of Surgery, Temple University School of Medicine, Philadelphia, PA 19140, USA

**Keywords:** DNA methylation, AAA, KLHL35, CNN2, SERPINB9, ADCY10P1, aortic aneurysm

## Abstract

Abdominal aortic aneurysm (AAA) is a complex disorder that has a significant impact on the aging population. While both genetic and environmental risk factors have been implicated in AAA formation, the precise genetic markers involved and the factors influencing their expression remain an area of ongoing investigation. DNA methylation has been previously used to study gene silencing in other inflammatory disorders and since AAA has an extensive inflammatory component, we sought to examine the genome-wide DNA methylation profiles in mononuclear blood cells of AAA cases and matched non-AAA controls. To this end, we collected blood samples and isolated mononuclear cells for DNA and RNA extraction from four all male groups: AAA smokers (*n* = 11), AAA non-smokers (*n* = 9), control smokers (*n* = 10) and control non-smokers (*n* = 11). Methylation data were obtained using the Illumina 450k Human Methylation Bead Chip and analyzed using the R language and multiple Bioconductor packages. Principal component analysis and linear analysis of CpG island subsets identified four regions with significant differences in methylation with respect to AAA: kelch-like family member 35 (*KLHL35*), calponin 2 (*CNN2*), serpin peptidase inhibitor clade B (ovalbumin) member 9 (*SERPINB9*), and adenylate cyclase 10 pseudogene 1 (*ADCY10P1*). Follow-up studies included RT-PCR and immunostaining for *CNN2* and *SERPINB9*. These findings are novel and suggest DNA methylation may play a role in AAA pathobiology.

## 1. Introduction

Abdominal aortic aneurysm (AAA) is a complex disease that develops due to the interaction of environmental risk factors and genetic predisposition [1,2,3,4]. In addition to an individual’s DNA sequence, other mechanisms can control gene expression, and influence the resulting phenotype of health or disease. The process of controlling gene expression through these alternative methods is known as epigenetics and includes RNA associated silencing, histone modifications and DNA methylation [5]. DNA methylation, the most well studied epigenetic modification, is a process in which a methyl group is added to a region where a cytosine nucleotide is located next to a guanine nucleotide that is linked by a phosphate. A cluster of CpGs is called a CpG island (CpGI) [5,6]. CpGIs are methylated by a group of enzymes called DNA methyltransferases. Classically, insertion of methyl groups at CpGIs was thought to block the binding of transcription factors to promoters and therefore result in repressed gene expression. More recent investigations have demonstrated that DNA methylation in other regions of the genome, including the gene bodies, are likely to influence gene expression, thus necessitating a comprehensive analysis of all DNA methylation sites [7,8,9].

Despite uncertainty in its precise role, there is accumulating evidence implicating DNA methylation in several common chronic human disease states such as cancer, diabetes, autoimmune disorders and atherosclerosis [10,11,12,13,14]. Cigarette smoking has proven to be a powerful environmental modifier of DNA methylation [15] and is a potential mechanism by which tobacco can affect gene expression. The role of smoking in the development of AAA is well known and a recent meta-analysis demonstrated that smoking is also associated with increased AAA growth and rupture risk [16,17]. Moreover, AAA in both man as well as animal models is characterized by an extensive involvement of the immune system with contributions from a variety of immune cell types [1,18,19,20]. Despite the potential relationship between cigarette smoking, AAA formation, inflammation and DNA methylation, no investigation has examined the role of DNA methylation in AAA. In this current study, we analyzed the genome-wide DNA methylation profile of patients with AAA using peripheral blood mononuclear cells (PBMC), which have been used for methylation studies in other inflammatory disorders such as inflammatory bowel disease, rheumatoid arthritis, systemic lupus erythematosis and Sjögren’s syndrome [21,22,23,24,25]. We hypothesize that alterations in DNA methylation are responsible for AAA development and progression.

## 2. Results

**Figure 1:**
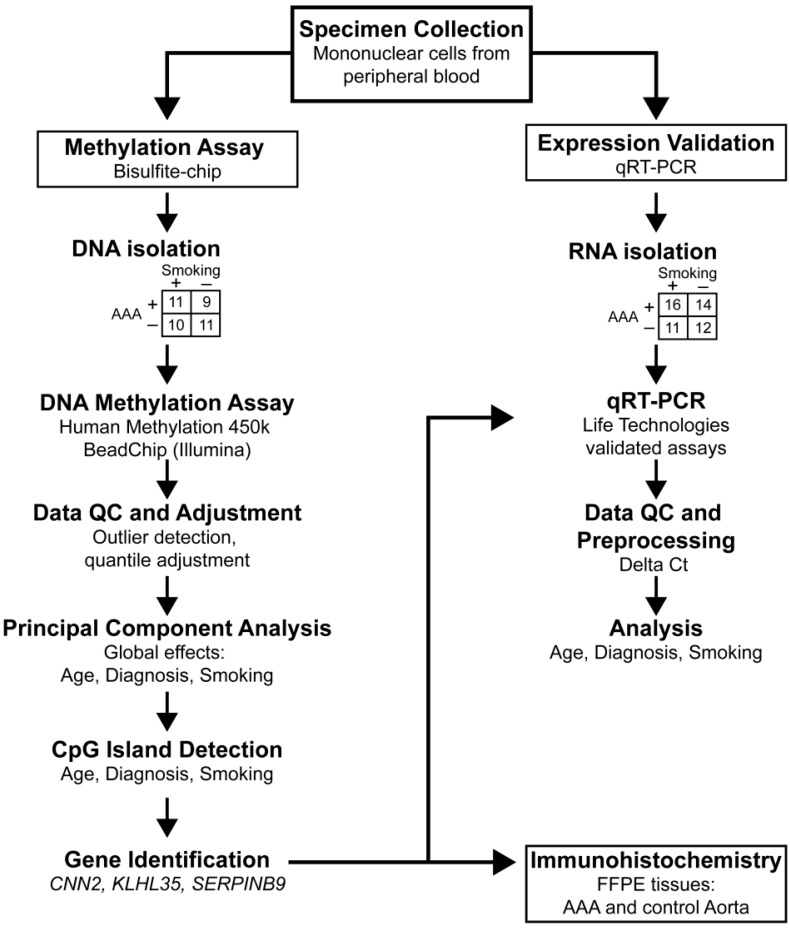
Outline of the study. We collected blood samples and isolated mononuclear cells for DNA and RNA extraction from four all male groups: AAA smokers, AAA non-smokers, control smokers and control non-smokers. Methylation data were obtained using the Illumina 450k HumanMethylation Bead Chip [26] and analyzed using the R language and multiple Bioconductor packages. Principal component analysis and linear analysis of CpG island subsets identified four regions with significant differences in methylation with respect to AAA: kelch-like family member 35 (*KLHL35*), calponin 2 (*CNN2*), serpin peptidase inhibitor clade B (ovalbumin) member 9 (*SERPINB9*), and adenylate cyclase 10 pseudogene 1 (*ADCY10P1*) (pseudogene). Follow-up studies included RT-PCR and immunostaining for *CNN2* and *SERPINB9*. Symbols: AAA, abdominal aortic aneurysm; FFPE, formalin-fixed paraffin-embedded tissue; QC, quality control.

We investigated changes in DNA methylation in patients with AAA and followed up the findings by conducting gene and protein expression studies. Figure 1 presents an outline of our study. The AAA patients included in the methylation study had aortic diameters between 3 and 5 cm and none of them had undergone a surgical AAA repair. First, genome-wide DNA methylation patterns were obtained from PBMC of AAA (*n* = 20) and non-aneurysmal (*n* = 21) patients. The DNA methylation data were analyzed by principal component (PC) analysis, and then a subset of CpGIs were further analyzed using ordinary least squares (OLS) linear regression models to detect significant differences in methylation. This analysis revealed four genes with significantly different DNA methylation in AAA samples compared with controls. Two genes with the greatest magnitude of differential methylation, calponin 2 (*CNN2*) and serpin peptidase inhibitor clade B (ovalbumin) member 9 (*SERPINB9*), were studied further using real-time quantitative RT-PCR to determine their mRNA levels in PBMC of AAA (*n* = 26) and non-aneurysmal (*n* = 20) controls. In addition, immunohistological staining of AAA (*n* = 6) and control human infrarenal abdominal aortas (*n* = 4) was used to determine protein expression and localization. All blood samples were obtained from male patients between the ages of 43 and 88 (Table 1 and Supplementary Table S1). Human aortic AAA samples for immunohistological staining were collected from patients undergoing AAA repair operations, and non-aneurysmal infrarenal abdominal aortic wall samples were collected from autopsies (Table 2).

**Table 1:** Summary of experimental groups used in the microarray-based DNA methylation and RT-PCR-based gene expression studies.

Experiment	Group	Smoking *	*n*	Age (Years ± SD)
DNA methylation (450k BeadChip)	Control	Yes	10	55.7 ± 8.0
	Control	No	11	67.0 ± 11.2
	AAA	Yes	11	65.8 ± 5.2
	AAA	No	9	77.3 ± 9.3
Gene Expression (Q-RT-PCR)	Control	Yes	10	58.0 ± 9.0
	Control	No	10	67.3 ± 13.0
	AAA	Yes	14	68.1 ± 4.8
	AAA	No	12	78.3 ± 6.1

* Subjects were considered non-smokers if they never smoked or did not smoke during the past 20 years. Subjects were considered smokers if they were current smokers. See Supplementary Table S1 for details on each donor. All donors were Caucasian males. The diameter of every AAA was between 3 and 5 cm and none of the AAA patients had undergone a surgical AAA repair. AAA: abdominal aortic aneurysm.

**Table 2:** Human aortic tissue samples used for immunohistochemical analysis.

Case ID	Age (Years)	Sex	Cause of Death	Smoking *	Classification
ME0503	54	Male	Cardiac arrest	Unknown	Control
ME0501	69	Female	Head trauma	Unknown	Control
ME0105	78	Male	Cardiac arrest	Unknown	Control
ME0505	59	Female	Cardiovascular	Unknown	Control
ELM0065	77	Male	NA	No	AAA
ELM0038	65	Female	NA	No	AAA
ELM0056	61	Male	NA	No	AAA
ELM0060	77	Male	NA	Yes	AAA
ELM0041	62	Male	NA	Yes	AAA
ELM0063	71	Male	NA	Yes	AAA

* Subjects were considered non-smokers if they never smoked or did not smoke during the past 20 years. Subjects were considered smokers if they were current smokers. The smoking status of the control samples is unknown. All samples were collected from the infrarenal abdominal aorta. All donors were Caucasian. NA, not applicable, since the sample was obtained during an AAA repair operation.

### 2.1. Genome-Wide Analysis of DNA Methylation in Peripheral Blood Mononuclear Cells of AAA Patients and Non-Aneurysmal Controls

Human Methylation 450k BeadChips [26] were used to detect DNA methylation in AAA (*n* = 20) and control (*n* = 21) samples, and methylation at CpGIs was analyzed. Table 1 summarizes the samples used, and Supplementary Table S1 provides more detailed information about each donor. The 41 samples were run in two batches. PC analysis revealed there were significant global differences in methylation between the two batches. There were also significant differences in methylation with respect to age and AAA diagnosis. When OLS linear regression models included smoking, the fit for age was improved, compared to models that did not include smoking.

Based on the PC analysis, a subset of 16 CpGIs contributed most to the variance of the PCs significant for AAA diagnosis and age (Table 3 and Supplementary Table S2). The CpGIs selected for the subset also had a similar direction and magnitude in both batches. This subset was analyzed using OLS linear regression models, and four CpGIs were found to be significantly differentially methylated with respect to AAA (Table 4). Each of these CpGIs are located in the body of a different gene. These genes were *ADCY10P1*, *CNN2*, *KLHL35*, and *SERPINB9*. DNA methylation was decreased in AAA samples compared to controls for *ADCY10P1* and *CNN2*. DNA methylation was increased in AAA samples compared to controls for *KLHL35* and *SERPINB9*.

**Table 3:** CpGIs in the subset analyzed using ordinary least squares (OLS) linear regression models.

CpGI * Name and Location	Gene	Gene Context of the CpGI
chr4: 190962111–190962689	null	NA
chr6: 2891929–2892182	*SERPINB9*	Body
chr6: 41068475–41069343	*ADCY10P1*	Body
chr6: 168435835–168436086	*KIF25*	Body
chr9: 124987743–124991086	*LHX6*	Body
chr11: 396685–397462	*PKP3*	Body
chr11: 75139454–75139817	*KLHL35*	Body, Promotera
chr17: 152117–152438	*RPH3AL*	Body
chr17: 19099818–19100138	null	NA
chr19: 1033605–1035236	*CNN2*	Body
chr19: 37786692–37787110	null	NA
chr19: 612989–614068	*HCN2*	Body
chr19: 49001748–49003087	*LMTK3*	Body
chr20: 32254811–32255989	*NECAB3*	Body
chr21: 38630052–38630507	*DSCR3*	Body
chrX: 72298626–72299108	*PABPC1L2A*	1st Exon

* The CpGIs were represented by 16 CpG probes and spanned both Promoter and Body of the gene. Only 3 of 16 probes were in the promoter. NA, not applicable, since the CpGI was not in any gene. Extended annotation for each CpG probe in the CpGIs above is provided in Supplementary Table S2.

**Table 4:** Genes demonstrating differential DNA methylation with respect to AAA, age, and smoking.

Gene	*p* Value (Diagnosis)	*p* Value (Age)	*p* Value (Smoking)
*ADCY10P1*	0.0221	0.06702	0.04543
*CNN2*	0.006514	0.032	0.02625
*KLHL35*	0.009153	0.009907	0.007803
*SERPINB9*	0.00309	0.02121	0.1016

### 2.2. Real-Time Quantitative RT-PCR to Measure mRNA Levels of CNN2 and SERPINB9 in PBMC of AAA Patients and Non-Aneurysmal Controls

Two of the genes (*CNN2* and *SERPINB9*) which were significantly differentially methylated in AAA were selected for follow-up studies which included real-time quantitative RT-PCR and immunohistochemical staining.

**Figure 2:**
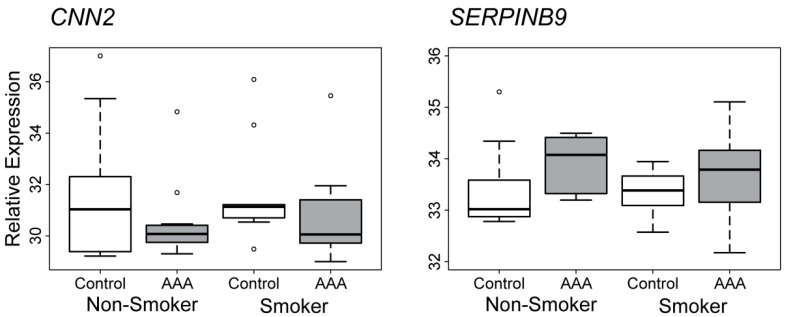
Comparison of *CNN2* and *SERPINB9* mRNA levels in PBMC of AAA smokers (*n* = 14), AAA non-smokers (*n* = 12), control smokers (*n* = 10) and control non-smokers (*n* = 10). The *C*_t_ difference was calculated by subtracting the *C*_t_ value of the *RPL* gene (housekeeping gene) from the *C*_t_ value of *CNN2* (or *SERPINB9*) for each sample. The difference was then subtracted from the value 40 (highest cycle count) to obtain values in which larger value means higher expression and lower value means lower expression level. Each sample was run in triplicate. Box-and-whisker plots are presented, in which the thick horizontal bars in the boxes indicate median values, boxes indicate interquartile range, whiskers indicate range of non-outlier values, and open circles indicate outliers less than 3 interquartile range units. Gene symbols available from the National Center for Biotechnology Information (NCBI; http://www.ncbi.nlm.nih.gov/) were used. See Table 1 and Supplementary Table S1 for details on the donors.

Real-time quantitative RT-PCR was used to measure mRNA levels of *CNN2* and *SERPINB9* in PBMC of AAA (*n* = 26) and non-aneurysmal (*n* = 20) samples. Table 1 summarizes the sample groups, and Supplementary Table S1 lists more detailed information about each donor. There were no statistically significant differences in gene expression with respect to AAA diagnosis, age, or smoking. However, there was a trend for *CNN2* to have lower expression, and *SERPINB9* to have higher expression in the AAA than control samples (Figure 2).

### 2.3. Immunohistological Staining of CNN2 and SERPINB9 in the Aortic Wall of AAA Patients and Non-Aneurysmal Controls

**Figure 3:**
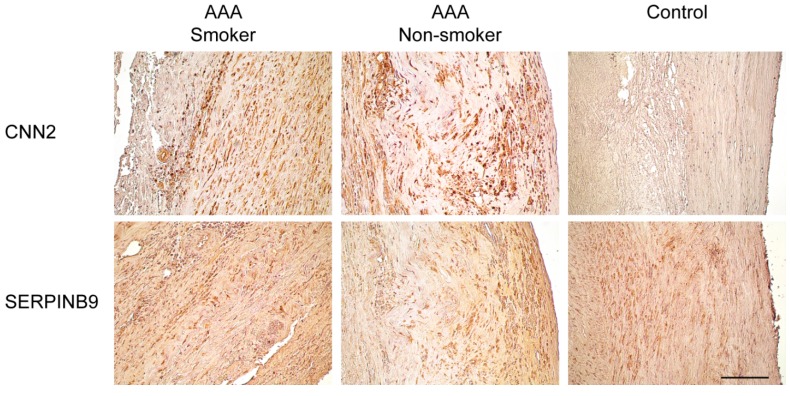
Immunohistochemical staining for CNN2 and SERPINB9 in human aneurysmal and non-aneurysmal abdominal aorta. See Table 2 and Table 5 for details on the aortic tissues and antibodies used, respectively. Scale bar = 100 μm.

Immunohistochemical analysis was performed to visualize expression and localization of *CNN2* and *SERPINB9* in the aortic wall of AAA (*n*_smoking_ = 3, *n*_non-smoking_ = 3) and non-aneurysmal (*n* = 4) patients. Table 2 includes information about each tissue sample used, and Table 5 includes information about the *CNN2* and *SERPINB9* primary antibodies used. Representative images of aortic tissue samples from a control, an AAA smoker, and an AAA non-smoker are shown in Figure 3. While imprecise at best, we attempted to compare the results of our immunohistochemical analysis. Subjectively, control aortas showed no specific staining for *CNN2* in any section of the aorta. AAA aortas displayed much more staining for *CNN2* throughout the media and intima. In samples containing a thrombus, the thrombus also contained many stained cells (not shown). There were no noticeable differences in staining between samples from smokers and non-smokers. Although a statistical comparison is not available, control aortas subjectively appeared to show *SERPINB9* staining in the intima and media. There was also light staining in the adventitia of some controls aortas, but it was not consistent. Female aortas contained less *SERPINB9* staining than male aortas (not shown). AAA aortas generally showed darker *SERPINB9* staining. This was especially noticeable near the intima of most samples. There was also an increase in *SERPINB9* staining in areas of the aorta where the morphology was disrupted and where there was a high density of immune cells.

**Table 5:** Primary antibodies used in immunohistological analyses.

Gene Symbol	Gene ID	Protein Symbols	Full Name	Catalog Number	Supplier	Species	IHC Dilution
*CNN2*	1265	CNN2	Calponin 2	TA503688	OriGene, Rockville, MD, USA	Mouse monoclonal	1:234
*SERPINB9*	5272	CAP3, PI9, SERPINB9	Serpin peptidase inhibitor, clade B (ovalbumin), member 9	TA312970	OriGene, Rockville, MD, USA	Rabbit polyclonal	1:4667

IHC, immunohistochemistry.

### 2.4 Bioinformatic Analysis of Interactions between CNN2 and SERPINB9

**Figure 4:**
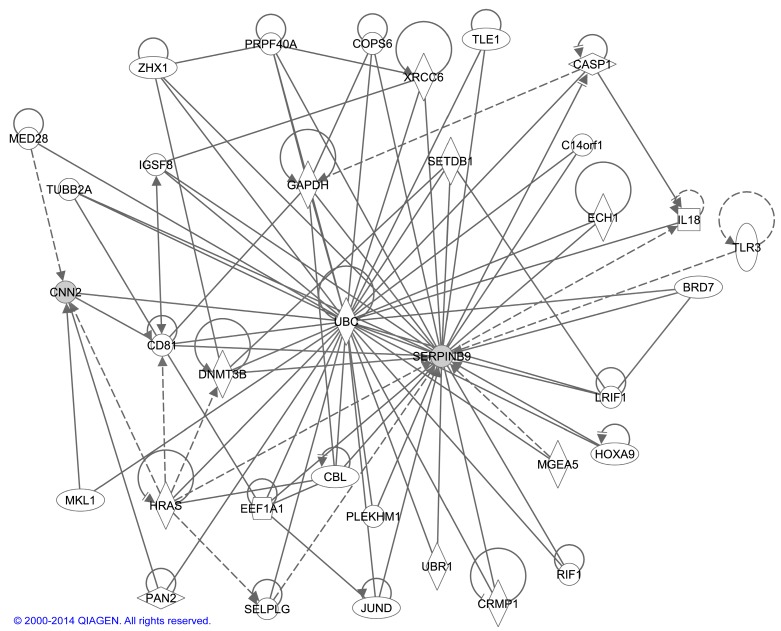
Network of interactions between *CNN2* and *SERPINB9*. Ingenuity Pathway Analysis^®^ (Qiagen’s Ingenuity Systems, Redwood City, CA, USA) tool was used for the analysis. Molecules are represented as nodes, and the biological relationship between two nodes is represented as a line. Solid lines represent direct interactions and dashed lines indirect interactions. All lines are supported by at least one literature citation or from canonical information stored in the Ingenuity Pathways Knowledge Base (Qiagen’s Ingenuity Systems). Nodes are displayed using various shapes that represent the functional class of the gene product.

Potential interactions between *CNN2* and *SERPINB9* identified using Ingenuity Pathway Analysis^®^ (IPA) tool are shown in a network (Figure 4). *SERPINB9* and *CNN2* do not directly interact, but they do interact with several of the same proteins and they are involved in similar pathways. Most of the genes in the network are involved in cell signaling, the immune response, and cell death.

## 3. Discussion

In this study, we examined the genome-wide DNA methylation in the PBMC of patients with AAAs. Since cigarette smoking and increasing age have been found to be an important mediator of DNA methylation in tobacco-related cardiovascular disease, it was necessary to take the age and the smoking history of the patients into consideration in the analyses [27]. Regardless of smoking status, our analysis demonstrated significant differences in DNA methylation at specific CpG islands that map to two genes of interest: *CNN2* and *SERPINB9*. While the precise functions of these genes remain under scrutiny, there is biologic plausibility to implicate these genes in AAA pathogenesis. The first gene is *CNN2*, which is also known as h2-calponin or calponin 2. CNN2 is an actin-binding protein implicated in cytoskeletal organization and vascular development [28]. Calponin has been found to be upregulated in stretched vascular walls and may be an important regulator of vascular smooth muscle cell phenotype [29]. Additionally, circulating levels of calponin are found in patients with acute aortic dissection and may be a potential biomarker for this aortic pathology [30]. The second gene is *SERPINB9*, which is also known as PI9. *SERPINB9* is located at 6p25 and belongs to a family of serine protease inhibitors. SERPINB9 is present in the cytoplasm of cytoxic lymphocytes, inhibits the granule proteinase granzyme B and protects cells from granzyme B induced apoptosis [31,32,33]. Moreover, SERPINB9 has been shown to inhibit apoptosis of human vascular smooth muscle cells, again a key process in AAA pathogenesis [34].

Our investigation began by investigating global DNA methylation in the PBMC of AAA patients (Table 1 and Supplementary Table S1). For comparison, we also analyzed global DNA methylation in the PBMC of vascular surgery patients without evidence of peripheral arterial disease (PAD) (Table 1 and Supplementary Table S1). We considered it important to use non-PAD patients as controls as others [8] have shown diffuse hypermethylation across many genomic loci in patients with systemic atherosclerosis. Our initial analysis, using Human Methylation 450k BeadChips [26], demonstrated decreased methylation of the *CNN2* gene and hypermethylation of the *SERPINB9* gene. Interestingly, none of the differentially methylated CpGIs within these genes were located in the promoter regions. These non-promoter CpGIs, regarded as CpGI shores, still influence gene expression but likely through a different mechanism such as alternative transcription or by maintaining silenced gene expression that was brought about by other epigenetic modifications [35,36]. Again, it is necessary to emphasize that the exact mechanism by which DNA methylation influences gene expression is still unknown. Regardless, we employed alternative methods to better understand the effects of DNA methylation on the genes of interest. First, we used real-time quantitative RT-PCR to examine the effect of methylation on gene expression. To our dismay, we found no difference in *CNN2* or *SERPINB9* mRNA in PBMC when comparing AAA and control patients. As a secondary method to reconcile these results, we subjected aneurysmal and non-aneurysmal human infrarenal aortic tissue to immunohistochemical analysis. Using this technique, we were able to convincingly demonstrate enhanced expression of CNN2 and SERPINB9 in the aneurysmal aorta, as compared to that of non-AAA controls. Of note, we found the greatest positive staining to be limited to the vascular smooth muscle cell rich media of the aneurysmal aorta (Figure 3). The significance of this is unclear but likely highlights the importance of the vascular smooth muscle cell, and its reaction to inflammation, in AAA pathogenesis. Our results, while somewhat discordant, mirror that of others [8,36] who have found that DNA methylation of CpGI shores functions in a complex manner to regulate gene expression in health and disease.

The findings of this study are novel but our investigation has several limitations that must be highlighted. The first is the small sample size associated with this study. This will be addressed in future studies as our research efforts continue and patient enrollment increases. Moreover, our future experiments will also include further characterization of the individual epigenetic marks using sequencing of bisulfite modified DNA and studies on histone modifications such as methylation and acetylation. The second limitation worthy of discussion is the possibility that the differential methylation seen in this study is age-related and not AAA-related, albeit we did attempt to adjust the analyses for age and smoking status. In this study, the control patients were on average ~10 years younger than their AAA counterparts (Table 1). Another limitation of this study is that we used all mononuclear cell types for our analysis. Isolating subtypes of mononuclear cells from peripheral blood has been performed by others [25,37] but is beyond the scope of this report. The last limitation applicable to our study pertains to any investigation of DNA methylation. By studying DNA methylation, we are investigating a genetic modification, the exact effect of which on gene expression is unknown making interpretation of differential methylation very difficult [38].

## 4. Experimental Section

### 4.1. Samples

#### 4.1.1. Processing of Blood Samples for DNA and RNA Isolation

Blood samples were collected from patients (*n* = 56) in BD Vacutainer^®^ CPT™ Cell Preparation Tubes (Becton, Dickinson and Company, Franklin Lakes, NJ, USA) at the Geisinger Medical Center, Danville, PA, USA. The collection of the samples was approved by the Institutional Review Board of Geisinger Clinic, Danville, PA, USA. Table 1 summarizes the groups used in the methylation and gene expression studies. All samples and information about the donors are listed in Supplementary Table S1. Patients were considered non-smokers if they never smoked or did not smoke in the last approximately 20 years. Patients who were current smokers were classified as smokers. Individuals who had given up smoking one day to 20 years ago were excluded from the study. All AAA patients were identified within our vascular surgery clinic and had a duplex ultrasound (DU) or computed tomography (CT) imaging demonstrating an aortic diameter >3 cm. All control patients were also identified within the same vascular surgery clinic. To qualify as a control, the subject must have had palpable pedal pulses (to exclude the inclusion of patients with peripheral arterial disease) and have had DU or CT imaging demonstrating an aortic diameter <3 cm. Patients who were <65 years of age or those without a smoking history also qualified for control status without radiographic confirmation of a normal aortic diameter provided the aortic pulse was normal upon physical examination by a vascular surgeon. These patients were deemed eligible for control status as AAA screening in this population is not currently covered by Medicare and is not recommended by the US Preventive Services Task Force [39].

PBMC were isolated from the blood samples within 1 h of the blood draw. The PBMC pellets were stored at −80 °C until used for the experiments. DNA was isolated from PBMC with DNeasy^®^ Mini Kit (catalog #69504; Qiagen, Valencia, CA, USA). RNA was isolated from PBMC using RNeasy^®^ Mini Kit (Qiagen). RNA was quantified using a NanoDrop (Thermo Fisher Scientific, Waltham, MA, USA). Quality of the RNA samples was assessed by 2100 Bioanalyzer (Agilent Technologies, Inc., Santa Clara, CA, USA) and RNA Integrity Numbers (RIN) are shown in Supplementary Table S1.

#### 4.1.2. Human Aortic Tissue Samples

Full thickness aortic wall tissue specimens were collected from patients undergoing AAA repair operations (*n* = 6) at the Geisinger Medical Center, Danville, PA, USA. Non-aneurysmal aortic samples (*n* = 4) were collected at autopsies. The same autopsy samples have been used in previous studies [40,41,42,43,44], and have demonstrated comparable results to aneurysmal tissue in mRNA and protein analyses. The collection of human tissues was approved by the Institutional Review Boards of Geisinger Clinic, Danville, PA, USA and Wayne State University, Detroit, MI, USA. Samples were stored in phosphate-buffered formalin and embedded in paraffin. Details on the samples are listed in Table 2.

### 4.2. Microarray-Based DNA Methylation Study

Human Methylation 450k BeadChips (Illumina, San Diego, CA, USA) were used to study methylation patterns in AAA and control samples using genomic DNA isolated from PBMC, as described above. The nature of the methylation sites assayed by the chips was extensively described recently [26]. Samples were run in two batches, and hybridizations were performed by HudsonAlpha Institute for Biotechnology (Huntsville, AL, USA). Table 1 summarizes the sample groups used and Supplementary Table S1 contains information about each donor.

Methylation data were analyzed using the R language and multiple Bioconductor packages [45,46,47,48,49,50]. Data were quantile adjusted. Methylation data from the chips are ratio beta values, which are calculated by the intensity of the methylated probe over the total intensity. Using the wateRmelon package, ratio β values were quantile adjusted. Then, ratio β values were converted to *M* values because ratio statistics are between 0 and 1, which does not work well in linear analyses. This conversion was done using the wateRmelon package [47]. The package transforms the values using this formula: *M* = log [β/(1 − β)]. PC analysis was performed for both batches combined to detect batch differences, and separately to detect global differences in methylation due to diagnosis, age, and smoking. By performing the PC analysis tests were performed on the reduced data without testing each CpGI as a separate hypothesis. We therefore did not correct the beadchip data for multiple testing. We also reduced false positives by using two separate data sets to identify CpGIs which were concordant between the two data sets.

Next, a subset of CpGIs was created. First, PCs significant for diagnosis, age, and smoking were chosen from each batch. Then the CpGIs that contributed most to the variance within those PCs were isolated. Finally, CpGIs that had the same direction and magnitude of change in each batch were selected, resulting in a subset of 16 CpGIs. This subset was analyzed using OLS linear regression models to determine relevance of diagnosis and covariates [49]. The IlluminaHumanMethylation450kanno. ilmn12.hg19 package from Bioconductor was used to annotate genes [48].

### 4.3. Real-Time Quantitative Reverse Transcriptase PCR

Validated commercially available gene expression assays for *CNN2* and *SERPINB9* (Applied Biosystems, Life Technologies Corporation, Carlsbad, CA, USA) were used to detect mRNA in AAA and control samples using RNA isolated from PBMC, as described above. Levels of 18S RNA and *RPL* mRNA were determined to standardize the results, and all experiments were run in triplicate. Table 1 summarizes the samples used and Supplementary Table S1 contains information about the donors. After PCR, baselines and threshold values were set for signals using Sequence Detection System software according to the manufacturer’s recommendations (Applied Biosystems) and the threshold cycle numbers (*C*_t_) were computed for each well. Relative expression levels were calculated using the ∆*C*_t_ method where the expression of *RPL* was an internal control. Statistical analysis was performed using OLS linear regression models as implemented in the program R [46]. The results are presented as a box-and-whiskers plot [51].

### 4.4. Immunohistochemical Analysis of Aortic Tissue

Immunostaining was carried out with formalin-fixed paraffin-embedded tissue as previously described [52]. The slides were incubated with a primary antibody (Table 5) on an automatic immunostainer (Autostainer; DAKO, Carpinteria, CA, USA). A secondary antibody with peroxidase labelled polymer (UltraVision LP Detection System, Thermo Fisher Scientific Lab Vision, Freemont, CA, USA) was used and the signal was detected with substrate chromogen solution. All antibodies were first tested on tissue known to contain the protein of interest as positive controls. Nonspecific IgG antibody in lieu of primary antibody served as a negative control.

For evaluation of the stained slides microscope Nikon Optiphot-2 (Tokyo, Japan) equipped with Nikon Digital Camera DS-Fi2 was used. The camera software was NIS-Elements V4.13 (Nikon).

### 4.5. Bioinformatic Analyses

Potential interactions between *CNN2* and *SERPINB9* were analyzed using Ingenuity Pathway Analysis^®^ (IPA) tool version 9.0 (Qiagen’s Ingenuity Systems, Redwood City, CA, USA; www.ingenuity.com). Results were presented as a network.

## 5. Conclusions

By examining genome-wide DNA methylation in the peripheral blood mononuclear cells from AAA patients, we have identified locus-specific alterations at two genes, *CNN2* and *SERPINB9*, with biologic plausibility. These findings are novel and should prove valuable to researchers investigating the mechanisms of cardiovascular disease.

## Supplementary Materials

Supplementary materials can be found at http://www.mdpi.com/1422-0067/16/05/11259/s1.
