# Supplementary material for: The Potential Role of DNA Methylation in Abdominal Aortic Aneurysms
Source: Int J Mol Sci. 2015 May 18;16(5):11259–75. doi: 10.3390/ijms160511259 (PMC4463699; doi:10.3390/ijms160511259)
Supplement: Supplementary file 1 [file ijms-16-11259-s001.pdf]

Supplementary Information

Table S1. Donor and sample information for blood samples used in the microarray and gene expression studies.

Sample ID	Age (Years)	Diagnosis	Smoking	Classification	DNA Methylation	Gene Expression	RIN
ER001	68	AAA	No	AAA	√	-	8.60
ER002	68	AAA	Yes	AAA	√	√	-
ER004	56	AAA	Yes	AAA	√	-	4.90
ER005	85	AAA	No	AAA	√	√	-
ER006	68	AAA	Yes	AAA	√	√	5.80
ER007	72	AAA	Yes	AAA	√	√	-
ER008	60	AAA	Yes	AAA	√	√	6.50
ER009	66	AAA	Yes	AAA	√	√	7.30
ER010	61	VV	No	Control	√	-	9.40
ER012	72	VV	No	Control	√	√	8.90
ER013	43	VV	Yes	Control	√	√	8.60
ER015	75	VI	No	Control	√	-	7.00
ER016	65	VV	Yes	Control	√	-	7.60
ER017	47	VV	No	Control	√	√	-
ER021	86	AAA	No	AAA	√	√	-
ER023	85	AAA	No	AAA	√	√	-
ER024	48	VV	No	Control	√	√	8.80
ER025	74	ESRD	No	Control	√	√	6.30
ER026	82	AAA	No	AAA	√	-	4.90
ER028	67	VTE	Yes	Control	√	√	3.20
ER029	58	Unknown	Yes	Control	√	√	9.10
ER030	50	DVT	Yes	Control	√	√	8.40
ER031	46	DVT	Yes	Control	√	-	8.50
ER032	60	VV	Yes	Control	√	√	8.50
ER033	62	VV	No	Control	√	√	8.50
ER034	69	AAA	Yes	AAA	√	√	8.50
ER035	56	Unknown	Yes	Control	√	√	8.70
ER036	68	AAA	Yes	AAA	√	√	8.50
ER038	71	AAA	Yes	AAA	√	√	8.00
ER039	61	CD	Yes	Control	√	√	9.10
ER040	67	AAA	Yes	AAA	√	√	8.60
ER041	80	CD	No	Control	√	√	9.30
ER042	77	AVM	No	Control	√	-	8.90
ER045	70	CD	No	Control	√	√	9.00
ER046	51	ESRD	Yes	Control	√	√	8.50
ER048	71	AAA	No	AAA	-	√	8.20
ER049	59	VV	Yes	Control	-	√	9.20
ER050	71	CD	No	Control	√	√	8.20
ER051	76	AAA	No	AAA	√	√	5.40
ER052	76	AAA	No	AAA	√	√	8.30
ER053	59	AAA	Yes	AAA	√	√	8.80
ER054	58	AAA	No	AAA	√	-	8.60

Table S1. *Cont.*

Sample ID	Age (Years)	Diagnosis	Smoking	Classification	DNA Methylation	Gene Expression	RIN
ER056	80	AAA	No	AAA	√	-	8.20
ER057	69	AAA	No	AAA	-	√	-
ER066	69	AAA	Yes	AAA	-	√	-
ER067	88	CD	No	Control	-	√	-
ER068	61	CABG	No	Control	-	√	-
ER072	74	AAA	No	AAA	-	√	-
ER073	75	CD	Yes	Control	-	√	-
ER074	86	AAA	No	AAA	-	√	-
ER075	72	AAA	Yes	AAA	-	√	-
ER076	81	AAA	No	AAA	-	√	-
ER077	78	AAA	No	AAA	-	√	-
ER078	67	AAA	Yes	AAA	-	√	-
ER079	78	AAA	Yes	AAA	-	√	-
ER080	73	AAA	No	AAA	-	√	-

AAA, abdominal aortic aneurysm; AVM, arteriovenous malformation; CABG, coronary artery bypass graft; CD, carotid disease; DVT, deep vein thrombosis; ESRD, end stage renal disease; RIN, RNA Integrity Number obtained from Agilent BioAnalyzer; VI, venous insufficiency; VTE, venous thromboembolism; VV, varicose veins. All controls were without aneurysmal disease at the time of recruitment. See Table 1 for summary of sample groups used for the analyses. This table only includes samples that passed quality control filters and were used in the final analysis for both the DNA methylation and gene expression studies.

Table S2. Annotation for individual CpGs contained in the CpGIs that contributed to the principal components.

CpGI Identifier	CpG Name	Gene Symbol(s)	Gene Context of CpG	CpGI Context	Chromosome	Nucleotide Position
chr4: 190962111–190962689	cg22288613	NA	NA	Island	chr4	190962249
	cg25886683	NA	NA	S_Shore	chr4	190962794
	cg27154432	NA	NA	S_Shelf	chr4	190966117
chr6: 2891929–2892182	cg19577958	*SERPINB9*	3'-UTR	N_Shelf	chr6	2888212
	cg05175540	*SERPINB9*	Body	N_Shore	chr6	2890674
	cg09046168	*SERPINB9*	Body	Island	chr6	2891973
	cg22376758	*SERPINB9*	Body	Island	chr6	2892050
	cg20726195	*SERPINB9*	Body	Island	chr6	2892148
	cg10863922	*SERPINB9*	Body	Island	chr6	2892150
	cg01345354	*SERPINB9*	Body	Island	chr6	2892152
	cg11296767	*SERPINB9*	Body	S_Shore	chr6	2894011
	cg15838413	*SERPINB9*	Body	S_Shelf	chr6	2894553
chr6: 41068475–41069343	cg03942922	*NFYA*	Body	N_Shelf	chr6	41064480
	cg04082020	*NFYA*; *LOC221442*	3'-UTR; TSS1500	N_Shore	chr6	41068009
	cg02728342	*NFYA*; *LOC221442*	3'-UTR; TSS1500	N_Shore	chr6	41068173
	cg02167203	*NFYA*; *LOC221442*	3'-UTR; TSS1500	Island	chr6	41068553
	cg25110423	*NFYA*; *LOC221442*	3'-UTR; TSS200	Island	chr6	41068646
	cg04346459	*NFYA*; *LOC221442*	3'-UTR; TSS200	Island	chr6	41068666
	cg20398880	*NFYA*; *LOC221442*	3'-UTR; TSS200	Island	chr6	41068722
	cg09580153	*NFYA*; *LOC221442*	3'-UTR; TSS200	Island	chr6	41068724
	cg06671660	*NFYA*; *LOC221442*	3'-UTR; TSS200	Island	chr6	41068741
	cg03644281	*NFYA*; *LOC221442*	3'-UTR; TSS200	Island	chr6	41068752
	cg00322698	*NFYA*; *LOC221442*	3'-UTR; Body	Island	chr6	41069048
	cg19085918	*NFYA*; *LOC221442*	3'-UTR; Body	Island	chr6	41069119
	cg07403317	*LOC221442*	Body	S_Shore	chr6	41070479
	cg02575768	*LOC221442*	Body	S_Shelf	chr6	41073142

Table S2. *Cont.*

CpGI Identifier	CpG Name	Gene Symbol(s)	Gene Context of CpG	CpGI Context	Chromosome	Nucleotide Position
chr6: 168435835–168436086	cg23823000	*KIF25*	Body	N_Shelf	chr6	168433191
	cg21477075	*KIF25*	Body	N_Shelf	chr6	168433436
	cg01134139	*KIF25*	Body	N_Shore	chr6	168435636
	cg24246628	*KIF25*	Body	Island	chr6	168435914
	cg08476511	*KIF25*	Body	Island	chr6	168435923
	cg12003941	*KIF25*	Body	Island	chr6	168436019
	cg18319852	*KIF25*	Body	S_Shore	chr6	168436099
	cg14316629	*KIF25*	Body	S_Shore	chr6	168436353
	cg04775383	*KIF25*	Body	S_Shelf	chr6	168439228
chr9: 124987743–124991086	cg02825412	*LHX6*	Body	N_Shore	chr9	124985756
	cg09723488	*LHX6*	Body	Island	chr9	124987896
	cg04282082	*LHX6*	Body	Island	chr9	124988720
	cg21213617	*LHX6*	Body	Island	chr9	124989241
	cg21469772	*LHX6*	Body	Island	chr9	124989294
	cg13571460	*LHX6*	Body	Island	chr9	124989337
	cg13862711	*LHX6*	Body	Island	chr9	124989915
	cg03363289	*LHX6*	Body	Island	chr9	124990165
	cg00142257	*LHX6*	Body	Island	chr9	124990276
	cg13817952	*LHX6*	Body	Island	chr9	124990456
	cg06866657	*LHX6*	Body	Island	chr9	124990754
	cg21237939	*LHX6*	TSS200	Island	chr9	124991047
	cg13648318	*LHX6*	TSS1500	S_Shore	chr9	124991408
	cg22254104	*LHX6*	TSS1500	S_Shore	chr9	124991432
chr11: 396685–397462	cg17840408	*PKP3*	Body	Island	chr11	396686
	cg25258098	*PKP3*	Body	Island	chr11	396794
	cg01794156	*PKP3*	Body	Island	chr11	397077

Table S2. *Cont.*

CpGI Identifier	CpG Name	Gene Symbol(s)	Gene Context of CpG	CpGI Context	Chromosome	Nucleotide Position
chr11: 75139454–75139817	cg06329735	*KLHL35*	Body	N_Shore	chr11	75139390
	cg05353869	*KLHL35*	Body	Island	chr11	75139544
	cg04231094	*KLHL35*	Body	Island	chr11	75139680
	cg10909185	*KLHL35*	Body	Island	chr11	75139736
chr17: 152117–152438	cg27565067	*RPH3AL*	Body	N_Shelf	chr17	149484
	cg25416149	*RPH3AL*	Body	N_Shelf	chr17	149488
	cg08900396	*RPH3AL*	Body	N_Shelf	chr17	149582
	cg19040474	*RPH3AL*	Body	N_Shore	chr17	151914
	cg04897931	*RPH3AL*	Body	N_Shore	chr17	152089
	cg06755596	*RPH3AL*	Body	Island	chr17	152189
	cg06571687	*RPH3AL*	Body	Island	chr17	152259
	cg17316718	*RPH3AL*	Body	Island	chr17	152308
	cg17193961	*RPH3AL*	Body	Island	chr17	152350
	cg08770870	*RPH3AL*	Body	S_Shore	chr17	154410
	cg11940040	*RPH3AL*	Body	S_Shore	chr17	154420
chr17: 19099818–19100138	cg27028682	NA	NA	Island	chr17	19100026
	cg10751605	NA	NA	Island	chr17	19100051
chr19: 612989–614068	cg06657917	*HCN2*	Body	Island	chr19	613111
	cg00587228	*HCN2*	Body	Island	chr19	613433
	cg17403731	*HCN2*	Body	Island	chr19	613505
	cg12159023	*HCN2*	Body	Island	chr19	613818
chr19: 1033605–1035236	cg10658703	*CNN2*	Body	N_Shore	chr19	1033242
	cg05220090	*CNN2*	Body	Island	chr19	1033606
	cg10730425	*CNN2*	Body	Island	chr19	1035093
	cg15975960	*CNN2*	Body	S_Shore	chr19	1035450

Table S2. *Cont.*

CpGI Identifier	CpG Name	Gene Symbol(s)	Gene Context of CpG	CpGI Context	Chromosome	Nucleotide Position
chr19: 37786692–37787110	cg22148999	NA	NA	N_Shore	chr19	37784800
	cg06862049	*LMTK3*	Body	Island	chr19	49001890
	cg18955367	*LMTK3*	Body	Island	chr19	49002338
	cg16194588	*LMTK3*	Body	Island	chr19	49002477
	cg26522157	*LMTK3*	Body	Island	chr19	49003082
	cg00861945	*LMTK3*	Body	S_Shelf	chr19	49007025
chr20: 32254811–32255989	cg01652532	*NECAB3*; *C20orf134*	Body; TSS1500	N_Shore	chr20	32253007
	cg06723459	*NECAB3*; *C20orf134*	Body; TSS1500	N_Shore	chr20	32253019
	cg12710480	*NECAB3*; *C20orf134*	Body; TSS200	N_Shore	chr20	32254216
	cg00478435	*NECAB3*; *C20orf134*	Body; 1st Exon	N_Shore	chr20	32254706
	cg07470512	*NECAB3*; *C20orf134*	Body; 1st Exon 5'-UTR	Island	chr20	32255052
	cg03904042	*NECAB3*; *C20orf134*	Body; 1st Exon	Island	chr20	32255491
	cg14921437	*NECAB3*; *C20orf134*	Body; 1st Exon	Island	chr20	32255988
	cg13403462	*NECAB3*; *C20orf134*	Body; 1st Exon 3'-UTR	S_Shore	chr20	32256071
chr21: 38630052–38630507	cg23737637	*DSCR3*	Body	N_Shelf	chr21	38627731
	cg01922450	*DSCR3*	Body	N_Shore	chr21	38628950
	cg05126444	*DSCR3*	Body	N_Shore	chr21	38629859
	cg11287055	*DSCR3*	Body	Island	chr21	38630234
	cg00628697	*DSCR3*	Body	Island	chr21	38630342
	cg18380783	*DSCR3*	Body	Island	chr21	38630506
	cg16704958	*DSCR3*	Body	S_Shore	chr21	38630728
	cg22515737	*DSCR3*	Body	S_Shelf	chr21	38632928
chrX: 72298626–72299108	cg08871618	*PABPC1L2A*	1st Exon 3'-UTR	N_Shore	chrX	72297591
	cg03505772	*PABPC1L2A*	1st Exon 5'-UTR	S_Shore	chrX	72299310

Annotations from IlluminaHumanMethylation450kanno.ilmn12.hg19 [48]. NA, not applicable, since the CpGI was not in any gene. UTR: Untranslated Regions.
